# Analysis of the spatiotemporal evolution of birth rates and influencing factors in the Yangtze River Basin

**DOI:** 10.1371/journal.pone.0316139

**Published:** 2024-12-31

**Authors:** Xiujuan Jiang, Qianhua Pan, Wei Xu, Jingyuan Sun, Sicheng Chen

**Affiliations:** 1 School of Civil Engineering and Architecture, Wuhan Institute of Technology, Wuhan, Hubei, China; 2 Infrastructure Construction Investment of China Construction Third Engineering Bureau Ltd, Wuhan, Hubei, China; China West University, CHINA

## Abstract

The declining birth rate is one of the world’s major challenges. There is much literature on birth rate research in China. However, there are few studies on spatial distribution and influencing factors of birth rate in the Yangtze River Basin. In this study, data from 11 regions of the Yangtze River Basin from 2006 to 2023 were used to analyze the spatial and temporal distribution characteristics of birth rates using GIS spatial visualization and four-quadrant diagram. At the same time, 13 factors affecting birth rates were combined to carry out research. The results show that: (1) In 2023, five regions reported birth rates above 7‰, with Tibet faring the best, while six regions had rates below 7‰, with Hunan being the least favorable. (2) The first type of birth rate area shows a process of slow increase—slight decrease—accelerated growth—rapid decrease; the second type of birth rate area shows a process of gradual decrease—moderate increase—rapid decrease—rapid increase—rapid decrease; the third type of birth rate area has increased rapidly since 2021. The three types of birth rate areas show the characteristics of the spatiotemporal pattern of continuous spread and development. (3) The aging rate, per capita GDP, proportion of primary industry output value, proportion of tertiary industry output value, female illiteracy rate, per capita disposable income, per capita consumption expenditure, urbanization rate, proportion of higher education, juvenile dependency ratio, and elderly dependency ratio have different degrees of influence on the birth rate.

## Introduction

Population is one of the major problems facing various regions in China. As one of the main factors affecting population growth, the birth rate has gradually become one of the research avenues for many scholars to try to solve the population decline. China’s family planning policy began in 1982, aiming to effectively control the population at that time. Its direct change was the rapid decline in population growth over the next 20 years. In 2011, to cope with the problems brought about by the rapid population decrease, China uninterruptedly implemented the " two-child policy for couples of which each partner is an only child "(2011), " two-child policy for couples of which one partner is an only child "(2013), "universal two-child policy " (2016) and "the third-child policy" (2021) during the period from 2011 to 2023 to increase the national population. However, the actual situation is that after the rebound of the birth rate in 2012, the birth rate began to decline sharply in 2017, and the birth rate level in 2022 has dropped to the lowest value of 6.77 ‰ since the founding of People’s Republic of China [1]. Zhou W (2023) mentioned that China’s birth rate will be lower than the population mortality rate around 2025, and China will usher in negative population growth [2]. The current birth rate in China is in a rapid decline, and the decline of the birth rate in natural basins, especially in the Great River Basin, cannot be ignored. As the first natural basin in China, studying the birth rate in the Yangtze River Basin is even more urgent.

Scholars have different perspectives on birth rates. In terms of spatial distribution: Foreign scholars’ research on the spatial pattern of the birth rate started early. John et al. (2001), by examining fertility preferences in various countries around the globe and observing the disparities in birth rates among nations, has identified that high fertility rates are predominantly found in South America, whereas low fertility rates are concentrated in Central America. [3]; Sobotka T (2017) proposed that there are obvious differences in fertility between developed and developing countries [4]; Monsa SS (2024) proposed that the continuous decline in world fertility has spread to the world from 1990 to 2015 with the study of global fertility [5]. Domestic scholars mainly focus on the spatial differentiation of provincial fertility. Wang DH et al. (2017) based on provincial panel data [6], Yang XH et al. (2018) based on cross-sectional data [7], Pang Y et al. (2022) combined with spatial panel data [8] Zhu RJ et al. (2024) based on the five-to-seven-census data and researched the spatial agglomeration of provincial birth rates and regional differences between provinces [9]. Liang G (2018) used the five-to-seven-census data to study the aging of counties in Hubei province [10]. Xu X et al. (2020) analyzed the spatial characteristics of urban aging with the urban population aging data from the sixth census in 2010 [11]. In terms of influencing factors, foreign scholars Cherry (2015) [12], Salvati L (2020) mentioned the impact of unemployment factors on fertility [13]; Oura P (2021) mentioned the urbanization rate on fertility[14], Sobotka T (2017) mentioned the impact of late childbearing on fertility [4]; Salamaliki et al. (2017) used Greece as the research object, proposing that income and consumption levels hurt fertility [15]; Lee J et al. (2020) used panel data provided by the Korea Institute of Public Finance (KIPF) as the research object. It is proposed that socioeconomic factors have a significant impact on fertility [16]; Hu G et al. (2021) took China as the research object, and proposed that education harms fertility [17], Yan L et al. (2023) proposed that housing price has a negative impact on birth rate and fiscal expenditure has a non-linear impact on birth rate [18]; Chinese scholars mainly focus on economic growth, population upbringing, housing prices, and cultural education levels in terms of fertility factors. Huang JX (2020) combined the birth rates and regional economic development levels of 31 provinces and cities in China in 2017, and through machine learning clustering analysis algorithms, concluded that the impact of economic development on birth rate is dynamic [19]; Yan Y et al. (2020), based on birth rate data from Beijing from 2000 to 2017, combined with regression analysis methods, concluded that Gross Domestic Product (GDP), the dependency ratio of children and adolescents, and the dependency ratio of the elderly are the main factors affecting the population birth rate with a positive relationship, while the level of education and the number of medical institutions have an inverse relationship with the birth rate. [20]; Yang XH (2018) [7] examined the population birth rates in China’s 31 provinces and cities over the period from 2001 to 2016. Applying a spatial regression model that incorporates spatial effects, the study found that the link between GNP and birth rates is not definitive, and that higher education levels are inversely associated with birth rates.; Ge YX (2015) proposed that urbanization would promote the decline of the fertility rate. [21]; Guo LZ(2022) took Hubei Province as the research object and selected a total of 14 indicators such as social, economic and forestry resources to analyze the influencing factors of the birth rate in Hubei Province [22], Yang GL (2023) proposed that the increase of fertility rate can be promoted by increasing per capita disposable income and introducing effective tax relief policies [23].

Overall, current research on fertility rates is spatially focused at the national [6, 7, 9], provincial [10, 22], and county [11] levels. However, There is a noticeable gap in identifying the spatiotemporal differentiation in birth rates along river basins. Regarding influencing factors, studies have primarily focused on unemployment rates, urbanization rates, delayed childbearing, resident income and consumption levels, the prevalence of higher education, and the costs associated with raising children and housing prices. However, academia has not reached a consensus on the significance of economic indicators, such as Gross National Product and disposable income of urban residents, on birth rates. This study focuses on 11 regions along the Yangtze River Basin as the research area. Using population birth rate data from 2006 to 2023, we employ GIS spatiotemporal analysis to reveal the characteristics of birth rate changes over time and space in the Yangtze River Basin. Breaking away from traditional administrative boundary constraints, we categorize regional birth rates into three types: low, medium, and high. We then conduct bivariate correlation analyses on influencing factors, including the controversial indicators such as Gross National Product and disposable income of urban residents. The aim is to provide a scientific basis for the joint control of population birth rates within the basin and for regional development.

## Materials and methods

### Variables and data sources

#### Provincial administrative boundaries, municipalities, and Yangtze River system vector data

The data comes from the National Geographic Information Public Service Platform (Sky Map) (https://www.tianditu.gov.cn). To help carry out the study of the birth rate along the Yangtze River Basin, the Institute refers to the Yangtze River Basin as the area through which the Yangtze River mainstream flows. The study covers the provinces and municipalities and involves two municipalities and eleven provinces. The two municipalities are Shanghai and Chongqing, and the nine provinces are Jiangsu, Anhui, Jiangxi, Hubei, Hunan, Sichuan, Yunnan, Qinghai, and Tibet.

#### Data sources

The data in this study are from the "China Statistical Yearbook" and "China Population and Employment Statistics Yearbook" over the years, and the time used is from 2006 to 2023 [1, 24, 25].

#### Variable selection and variable description

Referring to the experience of previous scholars, and taking into account the statistical changes of some data in the China Statistical Yearbook and the different starting times, the final variables were studied from the demographic, economic, and social levels, and 13 indicators were selected as the influencing factors of the population birth rate. See Table 1 for details. Subsequent impact factor indicators will be replaced with abbreviated letters in the table.

**Table 1:** List of influencing factor data.

variable	index	abbreviation	Indicator explanation
demographic factors	aging rate	AR	The proportion of population aged 65 and above in the total population
	Proportion of female population	PFP	The proportion of women in the total population
	urbanization rate	UR	The proportion of urban population in the total population
economic factors	per capita GDP	PCG	Gross domestic product per capita can reach in a certain period of time
	disposable income	DI	The average value of personal disposable income is often used to measure the living standards of a region.
	the per capita consumptive expenditure	PCCE	The total expenditure that residents spend to meet the daily consumption of their families is often used to measure the standard of living in a region.
	The proportion of production output value of the primary industry	PPOVOI	The proportion of the added value of the primary industry in GDP
	The proportion of production output value of the tertiary industry	PPOVTI	The proportion of the added value of the tertiary industry in GDP
social factors	Female illiteracy rates	FIR	The proportion of illiterate women aged 15 and over in the total population
	Proportion of higher education	PHE	The proportion of people aged 6 years and over with college degree or above in the population aged 6 years and over. The statistical caliber of the number of people with college degree or above in 2016 and beyond includes the demographic data of college, undergraduate and graduate students.
	Total dependency ratio	TDR	Ratio of non-working-age population to working-age population
	Proportion of children ’s upbringing	PCU	The ratio of the number of children to the number of working-age population in a region
	Proportion of elderly population dependency	PEPD	The ratio of the population aged 65 and above to the working-age population in a certain area

## Methods

### Birth rate classification

Combined with the 2006–2023 national birth rate and natural growth rate data, the 2022 and 2023 China Statistical Yearbook statistics birth rate data are 7.52 ‰ and 6.77 ‰, the same year natural growth rate is 0.34 and negative 0.6, which can be understood that when the national birth rate is at 7‰, the fertility replacement value of the national fertility rate appears. So this study divides the birth rate in the Yangtze River Basin into three types, the first type of birth rate: ≥10 ‰, the second type of birth rate: 7–10 ‰, and the third type of birth rate: ≤7 ‰.

### Bivariate correlation analysis

Correlation analysis studies the interrelationship between one variable and another, and the nature and tightness of the interrelationship between variables. In other words, the task of correlation analysis is to give a quantitative description of the correlation. According to the scores of the correlation coefficient, it can be divided into three types: weak correlation (0 to 0.33), medium correlation (0.33 to 0.67), and strong correlation (0.67 to 1). Bivariate correlation analysis mainly determines whether there is a correlation between the two variables through the Pearson correlation coefficient [26]. The data selected in this study are all quantitative variables (continuous variables). By testing two variables, it is found that they are in line with the normal distribution [27]. Therefore, the bivariate analysis method is suitable for the correlation analysis of the factors affecting the birth rate in this study.

## Results and analysis

### Temporal and spatial evolution of birth rates along the Yangtze River Basin

#### Interpretation of population birth rate data

(1) Interpretation of the Birth Rate and the Four-quadrant Chart of the Average Annual Growth Rate.

**Fig 1:**
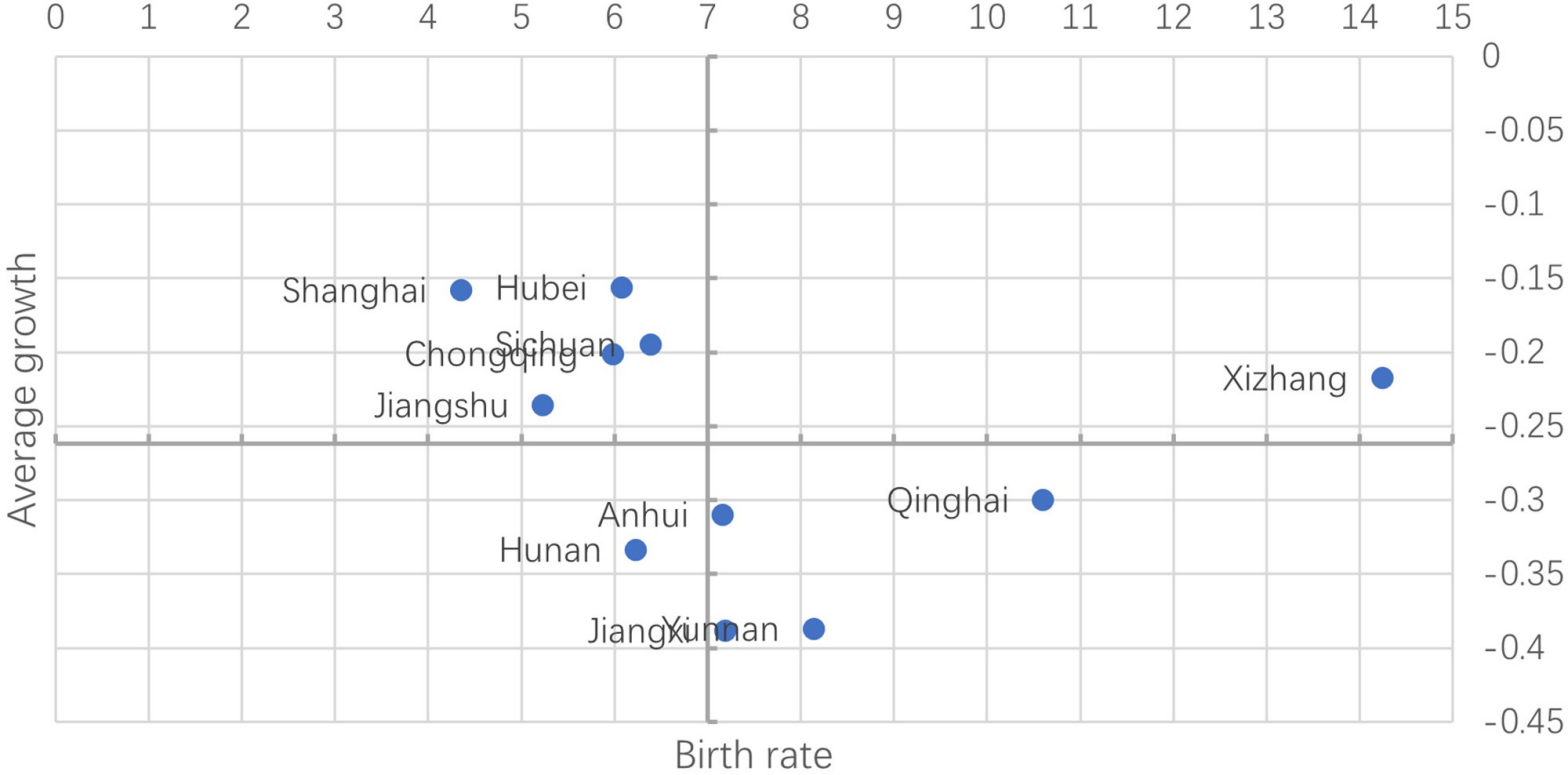
Quadrant chart of average annual growth rate.

The birth rate of 11 regions along the Yangtze River Basin in 2023 is taken as the horizontal axis, and the average annual growth rate of the birth rate from 2006 to 2023 is taken as the vertical axis to draw a scatter plot. To better analyze these 11 regions, the birth rate value of 7 ‰ was used as the horizontal axis 0 point, and the average annual growth rate of the birth rate of minus 2.88 was used as the vertical axis 0 point to redraw the scatter plot, and finally, it was plotted as the four-quadrant chart 1. The first quadrant is for areas where the birth rate is greater than 7 ‰ and the growth rate is greater than the average, and Tibet is the only one. The second quadrant is for areas where the birth rate is less than 7 ‰ but the growth rate is greater than the average, namely Shanghai, Hubei, Sichuan, Chongqing, and Jiangsu. The third quadrant is for areas where the birth rate is less than 7 ‰ and the growth rate is less than the average, and Hunan is the only one. The fourth quadrant is for areas where the birth rate is greater than 7 ‰ and the growth rate is less than the average. There are four regions, namely Qinghai, Anhui, Jiangxi, and Yunnan. From Fig 1, only five regions hold a birth rate greater than the fertility replacement rate in 2023, namely Anhui, Jiangxi, Yunnan, Tibet, and Qinghai. The average annual growth rate of the birth rate is the largest in Hubei (-0.15647) and the minimum in Jiangxi (-0.38824). The birth rate in the four quadrants has the greatest impact on the third quadrant area, where the fertility rate is less than 7 ‰ and the decline rate of the birth rate is less than the average annual rate.

#### Temporal and spatial changes in the birth rate of the three types of population

**Fig 2:**
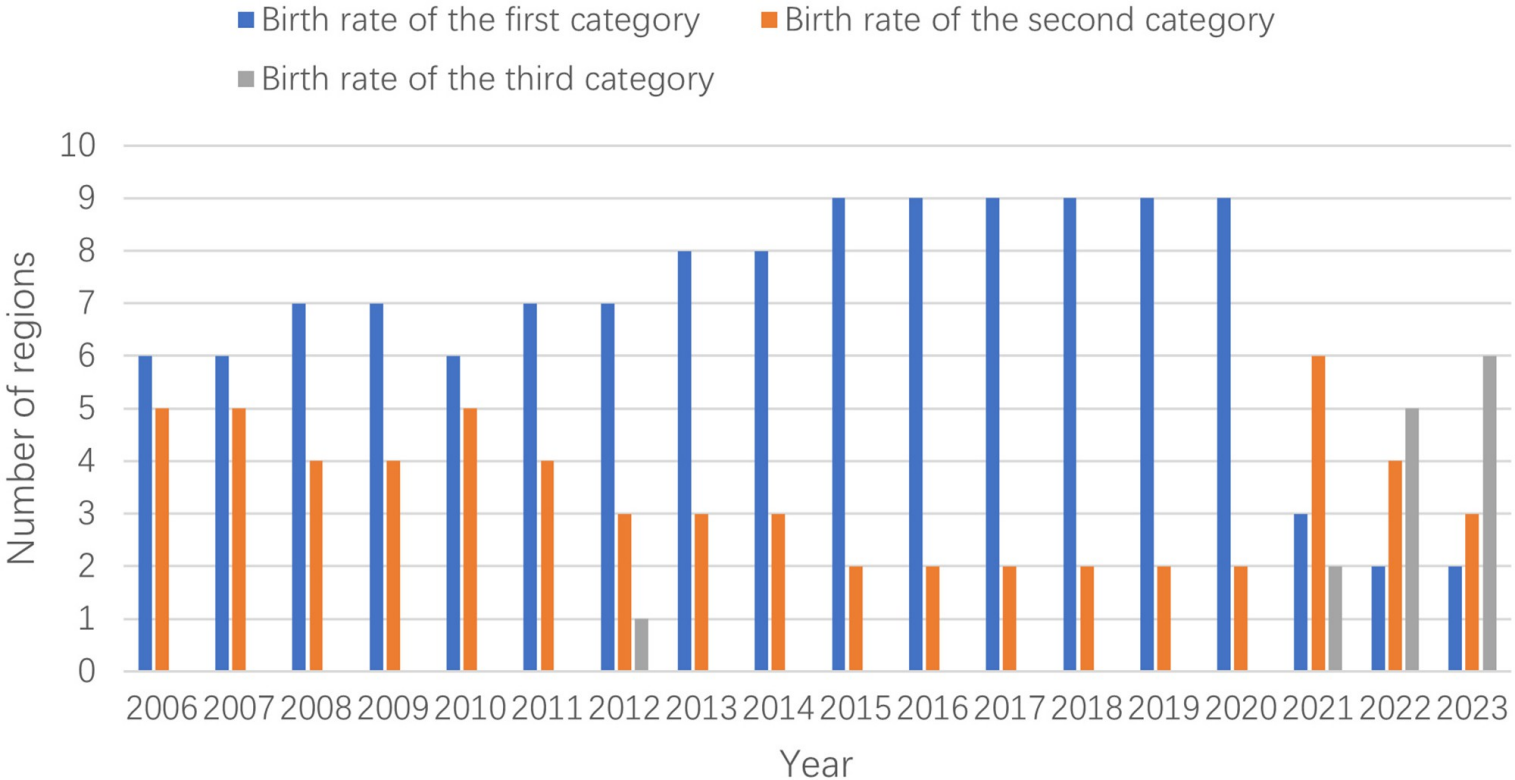
Number of birth rate regions in three categories (2006–2023).

The number of areas of various types from 2006 to 2023 is calculated and plotted in Fig 2, combining the three classification types of birth rate. It can be seen that the number of birth rate areas in the first category was rising before 2010, and the corresponding number of birth rate areas in the second category was declining. By 2010, the number of birth rate areas in the first category began to decline, but it began to rebound after 2011. The number of birth rate areas in this period kept rising and flat. This phenomenon should be related to China’s uninterrupted implementation of the birth policy during these years. Although China implemented the "three-child policy" in 2021, the number of birth rate areas in the first category showed a cliff-like decline in 2021, and the corresponding number of birth rate areas in the second category began to surge. The number of birth rate areas in the third category began to increase, and even in 2023. The number of birth rate areas in the third category was as high as six.

### Analysis of spatiotemporal characteristics

**Fig 3:**
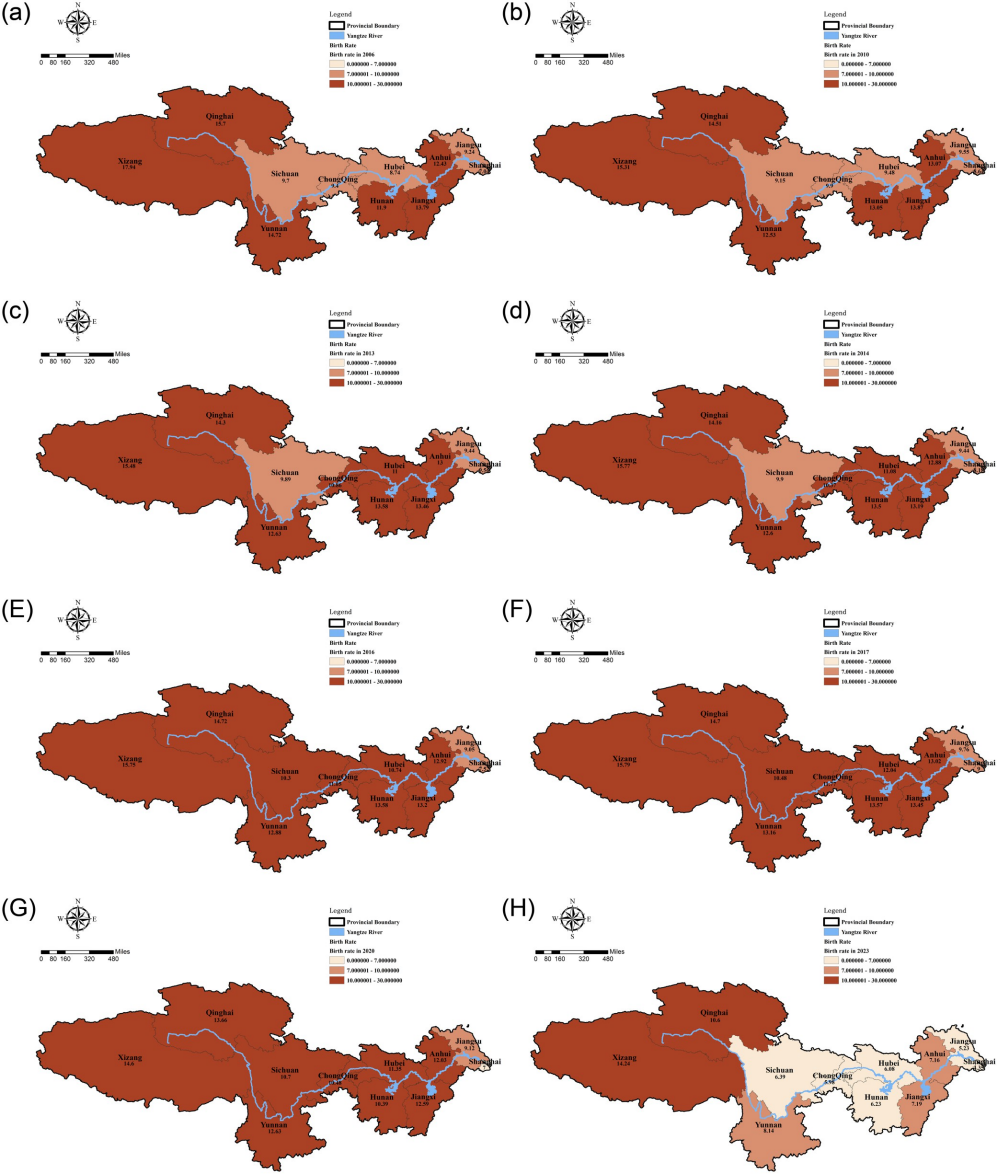
Spatial and temporal distribution of the three types of birth rate areas in the Yangtze River Basin. Note: 1. The administrative boundary map is based on the standard map of (Review No.: GS (2024) 0650) downloaded from the National Geographic Information Public Service Platform (Sky Map) (https://www.tianditu.gov.cn) of the Ministry of Natural Resources, and the boundary of the base map is not modified. 2. The Yangtze River Basin hydrological map is sourced from Open Street Map, a community of open-source map data. (https://www.openstreetmap.org/).

Combined with the three types of birth rate classification methods, and considering that China implemented the " two-child policy for couples of which each partner is an only child " in November 2011 to promote the number of nationals, the " two-child policy for couples of which one partner is an only child " in November 2013, the " universal two-child policy " in January 2016, and the three-child policy in May 2021. These policies will have a certain impact on the birth rate. At the same time, China’s Statistical Yearbook data are general statistics on the data at the end of the previous year. Therefore, combined with the birth rate data from 2006–2023, a total spatial and temporal distribution map of the birth rate in 2006, 2010, 2013, 2014, 2016, 2017, 2020, and 2023 was finally drawn (Fig 3).

Combined with the spatiotemporal distribution characteristics of the three types of birth rate areas, it can be seen that the birth rate areas of the same type tend to develop contiguously during the same period, but they are also affected by the Yangtze River system in terms of spatiotemporal distribution.

#### Spatiotemporal distribution of the first category birth rate region

In terms of time and space, it is characterized by "slow increase—slight decrease- accelerated growth—rapid decrease". From the two time points of 2006–2010, the birth rate values in various regions showed a slow increase during this period, but the number of birth rate areas and regions in the first category remained unchanged; the liberalization of the universal two-child policy in 2013 provided an opportunity for the slow growth of the birth rate. During this period, the birth rate in 11 regions in the Yangtze River Basin increased, especially in Hubei and Chongqing, where the birth rate exceeded 10 ‰, and even the birth rate value in Hubei exceeded 11 ‰; in 2016, The universal two-child policy began to be implemented. During this period, the birth rate in other regions of the Yangtze River Basin, except Shanghai and Jiangsu, exceeded 10 ‰, but this favorable policy lasted only a few years. By 2020, the birth rate began to decline in multiple regions. Although the first category of birth rate regions was still in contiguous development, compared to the previous period, the birth rate values in these regions began to decline; by 2021, less than half of the western part of the Yangtze River basin is the first category birth rate, and the number of the first category of birth rate areas in this period has declined rapidly. In 2022, only two regions in the western part of the Yangtze River Basin (Tibet and Qinghai) remained in the first category of birth rate areas. Although the spatial distribution of the first category of birth rate areas in 2023 is the same as in 2022, from the perspective of the remaining birth rate values in Tibet and Qinghai, the birth rate data in both regions have declined compared to 2022.

#### Spatiotemporal distribution of the second category birth rate regions

The second type of birth rate area is characterized by “gradual decrease—moderate increase—rapid decrease—sharp increase—rapid decrease” in time and space. From the two time points of 2006–2010, there was no change in the temporal and spatial distribution of the second category of birth rate areas, but most of them showed a slight upward trend in terms of the specific birth rate values of the areas; with the implementation of the "two-child policy for couples of which one partner is an only child " in 2013, the number of the second category of birth rate areas in 2013 and 2014 was reduced from the original five to three; the " the universal two-child policy" in 2016 injected a stronger force into the second category of birth rate areas. In 2016, compared with 2017, the number of birth rate areas in the second category decreased to only two; in 2020, the global birth rate showed a downward trend, and only Jiangsu remained in the second category; with the further decline of the global birth rate, by 2021, the number of birth rate areas in the second category soared to 7, accounting for nearly 65% of the number of areas in the Yangtze River Basin; in 2022, the number of birth rates in the whole region further decreased, and the second category of birth rate areas partially crossed the third category of birth rate, and some of the first birth rate areas began to enter the second type of birth rate, during this period, the number of the second type of birth rate areas reached 4; in 2023, the second type of birth rate areas began to break the spatial pattern of the original contiguous layout and began to show a point-like spatial pattern, during this period, the number of the second type of birth rate areas was 2.

#### Spatiotemporal distribution of the third category birth rate regions

The third type of birth rate area is characterized by "rapid development" in terms of time and space. The emergence of the third category of birth rate areas mainly began in 2020. During this period, the number of the third category of birth rate areas was only in Shanghai, but by 2021, the third category of birth rate areas began to spread outward with Shanghai as the center. By 2022, the third category of birth rate areas had begun to spread across Anhui to the northern side of the Yangtze River Basin, and the number of the third category of birth rate areas during this period was 6. In 2023, the third category of birth rate areas had begun to spread across the Yangtze River Basin to the southern side of the Yangtze River, and the number of the third category of birth rate areas during this period had reached 7, accounting for nearly 65% of the Yangtze River Basin area.

### Influencing factor analysis

The bivariate correlation analysis was conducted in combination with the birth rate of the first category, the birth rate of the second category, and the birth rate of the third category from 2006 to 2023. Combined with the Pearson correlation coefficient, the significant (two-tailed) number, and the number of samples of the birth rate and other 13 influencing factors indicators, the estimated results of the three samples were obtained (Table 2).

**Table 2:** Correlation analysis of three samples.

index	Birth rate of the first category	Birth rate of the second category	Birth rate of the third category
AR	-.620**	-.477**	-.407*
PFP	-.223**	-.457**	-.670**
UR	non-significant	.396**	.528**
PCG	non-significant	-.609**	non-significant
DI	non-significant	.400**	non-significant
PCCE	.403**	.478**	non-significant
PPOVOI	-.263**	-.505**	-.648**
PPOVTI	-.243**	-.528**	-.670**
FIR	-.410**	-.514**	-.536**
PHE	-.272**	-.592**	-.604**
TDR	non-significant	non-significant	non-significant
PCU	.485**	non-significant	.451*
PEPD	-.593**	-.412**	non-significant
sample size	123	61	14

**. At the 0.01 level (two-tailed), the correlation was significant.

*. At the 0.05 level (two-tailed), the correlation was significant.

#### Demographic factors

The aging rate shows a very significant negative correlation with the birth rate, which means that the higher the aging rate, the lower the birth rate. The proportion of women is very significantly positively correlated with the birth rate when the birth rate value is between 7–10‰, but in areas with a birth rate greater than or equal to 10‰ and less than 7‰, the birth rate is not significantly correlated with the proportion of women. In terms of urbanization rate, the birth rate value shows a very significant negative correlation with the urbanization rate, and with the increase of urbanization rate, the birth rate and its significance are further strengthened.

#### Economic factors

The per capita GDP output shows a very significant negative correlation with the birth rate in the three types of birth rates, and with the increase of the per capita GDP output, the birth rate will be further reduced. The proportion of the primary industry output value has no significant correlation with the birth rate value in the areas where the birth rate is relatively high, but in the areas where the birth rate is less than 10 ‰, it shows a very significant positive correlation with the birth rate, and the relationship with the birth rate is more significant when the birth rate falls below 7‰. The proportion of the tertiary industry output value shows a very significant negative correlation with the birth rate when the value of the birth rate region is between 7–10‰, and there is no significant correlation with the birth rate in other periods. Combined with the Pearson correlation coefficients of per capita disposable income and per capita consumption expenditure, these values show a significant negative correlation with the birth rate.

#### Social factors

The female illiteracy rate is extremely significantly positively correlated with the birth rate when the value of the birth rate area is greater than or equal to 7 ‰, but there is no significant correlation between the female illiteracy rate and the birth rate when the birth rate is less than 7‰; the proportion of higher education and the birth rate is extremely significantly negatively correlated, which reflects that with the improvement of higher education, the marriage age of students is postponed, the fluctuation of the conception, the original "more children and more blessings" and "raising children to prevent old age" ideas, helped reduce the fertility rate; the total dependency ratio did not have a significant impact on the birth rate, but the dependency ratio of children and adolescents showed a very significant positive correlation in areas with a birth rate greater than or equal to 10‰ and less than 7‰. This should have a great relationship with everyone’s conception and cognition of child-rearing. The dependency ratio of the elderly population in areas with a birth rate greater than 7‰ showed a very significant relationship with the birth rate, which also reflects the increase in the dependency ratio of the elderly population in one way or another, increasing the pressure on the social labor force, and further leading to a higher aging rate and a lower birth rate.

## Conclusions and discussion

Combined with the population birth rate values of 11 regions in the Yangtze River Basin from 2006 to 2023, according to the GIS spatial visualization, the spatial and temporal characteristics of the birth rate were analyzed, combined with the bivariate correlation analysis method, the influencing factors of the areas along the Yangtze River Basin were explored, and the conclusions were reached.

First of all, Birth rate data interpretation. Through the study of the national birth rate values, it was found that the birth rate reached the level of fertility replacement when the birth rate reached 7 ‰. Therefore, the birth rate in 2023 was the horizontal axis, and the origin of the horizontal axis was 7 ‰. The average annual growth rate of the birth rate in 11 regions from 2006 to 2023 was the vertical axis, and the average annual growth birth rate was the vertical axis. The four-dimensional quadrant scatter plot was constructed. Through the scatter plot, it was found that the birth rate in Hunan Province was poor both in terms of the birth rate value in 2023 and the annual growth rate of the birth rate in 2006–2023. In addition, the overall situation in Tibet is good. Although the birth rate in Qinghai, Anhui, Jiangxi, and Yunnan is more than 7 ‰ in 2023, the average annual decline in the birth rate in these areas is extremely fast, so the birth rate in these areas is likely to fall below 7 ‰ in the next few years.

Secondly, Temporal and spatial characteristics of the birth rate. In time and space: the first type of birth rate area showed a slow increase—slight decrease—acceleration of growth—rapid decline process; the second type of birth rate area showed a gradual decrease—moderate increase—rapid decrease—rapid increase—rapid decrease process; the third type of birth rate area time mainly began in 2021, and then increased rapidly. In terms of spatial layout, the first type of birth rate area mainly shows the spatial layout of contiguous development, and its development has experienced the development of contiguous development on the southwestern side of the Yangtze River Basin—across the north side of the Yangtze River Basin and in the middle of the west of the Yangtze River Basin—concentrated in the spatial layout of continuous development on the west side of the Yangtze River Basin; the second type of birth rate area is also dominated by the spatial layout of contiguous development, and its development has experienced the development of the two-point layout on the north side of the Yangtze River Basin—the single-point layout on the east side of the Yangtze River Basin—the development process of contiguous development on the east and north side of the Yangtze River Basin; the third type of birth rate area shows the development process of transformation from point-like space to sheet-like spatial layout.

Finally, Analysis of influencing factors. The aging rate, per capita GDP, proportion of primary industry output value, proportion of tertiary industry output value, female illiteracy rate, per capita disposable income, per capita consumption expenditure, urbanization rate, proportion of higher education, child-rearing ratio, and elderly population rearing ratio have different degrees of influence on the birth rate. Among them, the aging rate, per capita GDP, female illiteracy rate, per capita disposable income, per capita consumption expenditure, urbanization rate, proportion of higher education, elderly population dependency ratio, and birth rate showed a very significant negative correlation, the proportion of primary industry output value, female illiteracy rate, child dependency ratio and birth rate showed a significant positive correlation.

This study took eleven areas along the Yangtze River Basin as the research object, covering a time span from 2006 to 2023. This time frame not only ensures the timeliness of the data but also enriches the study of birth rates in the basin., but the research content did not deepen to the city and county level along the Yangtze River Basin, which makes it easy to ignore the county and city aspects due to the provincial average problem. Further deepening of the research content can be considered in the follow-up study. In this study, the research on the influencing factors of birth rate was mainly carried out from the demographic, economic, and social levels(see Table 1), and no in-depth research on the natural environment and location was carried out. In the subsequent research, we can try to start from natural environmental aspects such as topography and river systems, as well as from the locational aspects of economic and transportation positioning research on the influencing factors of the birth rate.

## Supporting information

S1 Table Relevant data underlying the findings described in manuscript. (XLSX)
